# Potassium fertilisation reduces radiocesium uptake by Japanese cypress seedlings grown in a stand contaminated by the Fukushima Daiichi nuclear accident

**DOI:** 10.1038/s41598-017-15401-w

**Published:** 2017-11-15

**Authors:** Masabumi Komatsu, Keizo Hirai, Junko Nagakura, Kyotaro Noguchi

**Affiliations:** 1 Department of Mushroom Science and Forest Microbiology, Forestry and Forest Products Research Institute, Tsukuba, Ibaraki, 305-8687 Japan; 2 Department of Forest Soils, Forestry and Forest Products Research Institute, Tsukuba, Ibaraki, 305-8687 Japan; 3 Tohoku Research Center, Forestry and Forest Products Research Institute, Morioka, Iwate, 020-0123 Japan

## Abstract

We analysed suppressive effects of potassium (K) fertilisation on radiocesium (^137^Cs) uptake by hinoki cypress (*Chamaecyparis obtusa*) seedlings from soils contaminated after the Fukushima Daiichi Nuclear Power Plant accident. Three-year-old seedlings were planted in a clear-cut forest (ca. 4 ha) during June–July 2014, and potassium chloride fertiliser (83 kg K ha^−1^) was applied twice (August 2014 and April 2015). ^137^Cs concentrations in the needles in the fertilised plots were one-eighth of those in the control (unfertilised) plots at the end of the second growing season (October 2015). Our results clearly indicated that K fertilisation reduced radiocesium transfer from soil to planted cypress seedlings. A linear mixed model analysis revealed that ^137^Cs concentrations in the needles were significantly affected by ^137^Cs inventory in the soil (Bq m^−2^) adjacent to the sampled seedlings, exchangeable K concentrations in surface mineral soils (0–5 cm) and fertilisation. The exchangeable K concentrations in surface soils in October 2015 did not differ from those in August 2014 (before fertilisation) in the fertilised plots and in the control plots. These results suggested that the levels of exchangeable K would temporarily increase by fertilisation during the growing season, and radiocesium uptake by tree roots was suppressed.

## Introduction

After the Fukushima Daiichi Nuclear Power Plant (FDNPP) accident following the large earthquake on 11 March 2011, large areas of forest lands, as well as other lands, were contaminated with the released radionuclides around the coastal and central areas of Fukushima prefecture. Among the radionuclides, cesium-137 (^137^Cs), one of the most released radionuclides, has a long half-life (about 30.2 years). Although ^137^Cs outflow flux from the forests was small^1^, ^137^Cs is considered to circulate within the forests^2^, and prolonged contamination of the forests is a great concern in this region. Several studies have been conducted for understanding the dynamics of radiocesium within the contaminated forests after the accident^3–5^. However, the transfer of radiocesium to the trees planted by afforestation after the accident has not been well studied, despite such information being indispensable to discuss future availability of wood and forest resources.

Radiocesium transfer to trees has been evaluated by the aggregated transfer factor (*T*_ag_, m^2^ kg^−1^)^6^, which is a ratio of radionuclide activity (Bq kg^−1^) of living organisms or their compartments to the radionuclide inventory per ground area (Bq m^−2^). The *T*_ag_ of tree compartments is defined by the following equation (1):

1 $${T}_{{\rm{ag}}}({{\rm{m}}}^{2}{{\rm{kg}}}^{-1})=\frac{{\rm{Activity}}\,{\rm{concentration}}\,{\rm{in}}\,{\rm{tree}}\,{\rm{compartments}}\,(\text{Bq}\,{{\rm{kg}}}^{-1})}{{\rm{Total}}\,{}^{137}{\rm{C}}{\rm{s}}\,{\rm{inventory}}\,{\rm{of}}\,{\rm{soil}}\,(\text{Bq}\,{{\rm{m}}}^{-2})}$$

The *T*_ag_ is used for natural or semi-natural environments, including forests, since vertical and horizontal distribution of radionuclides is highly heterogeneous in such environments. In this study, we define the total soil ^137^Cs inventory as sum of ^137^Cs inventory of organic layer and that of mineral soil from 0 cm to 20 cm depth.

Such *T*_ag_ concept intends that the radiocesium transfer to trees would be usually controlled by radiocesium absorption from soils via roots. However, recent studies have shown that not only root absorption but also foliar uptake and translocation of radiocesium deposited on the leaf surface might play an important role in the initial contamination of the internal parts of trees^7–9^. In addition, the initially contaminated radiocesium in woods would remain within the trees for a long time^10^. Therefore, to reduce radiocesium levels of forest trees in the contaminated area, it would be effective to grow newly planted trees with no initial contamination, and understanding the radiocesium dynamics in such planted trees is also necessary for future forestry in the contaminated areas.

The transfer of radiocesium via roots is affected by soil environmental conditions and tree species^11^. In particular, potassium (K) present in the soil is considered as a key factor, because cesium is an alkali metal, and K and the K transporters in plant roots could probably affect the uptake of cesium, especially under low external K concentrations^12^. Furthermore, it has been reported that K fertilisation in paddy fields and farmlands reduced radiocesium transfer to rice and other crop plants growing on the soil contaminated by the FDNPP accident^13,14^.

In the case of trees, some reports show that K fertilisation reduced the radiocesium activity of trees (pine) after the Chernobyl Nuclear Power Plant (ChNPP) accident in 1986^15,16^. However, to our knowledge, no study has reported about the effects of K fertilisation on the radiocesium activity of forest trees after the FDNPP accident.

**Figure 1:**
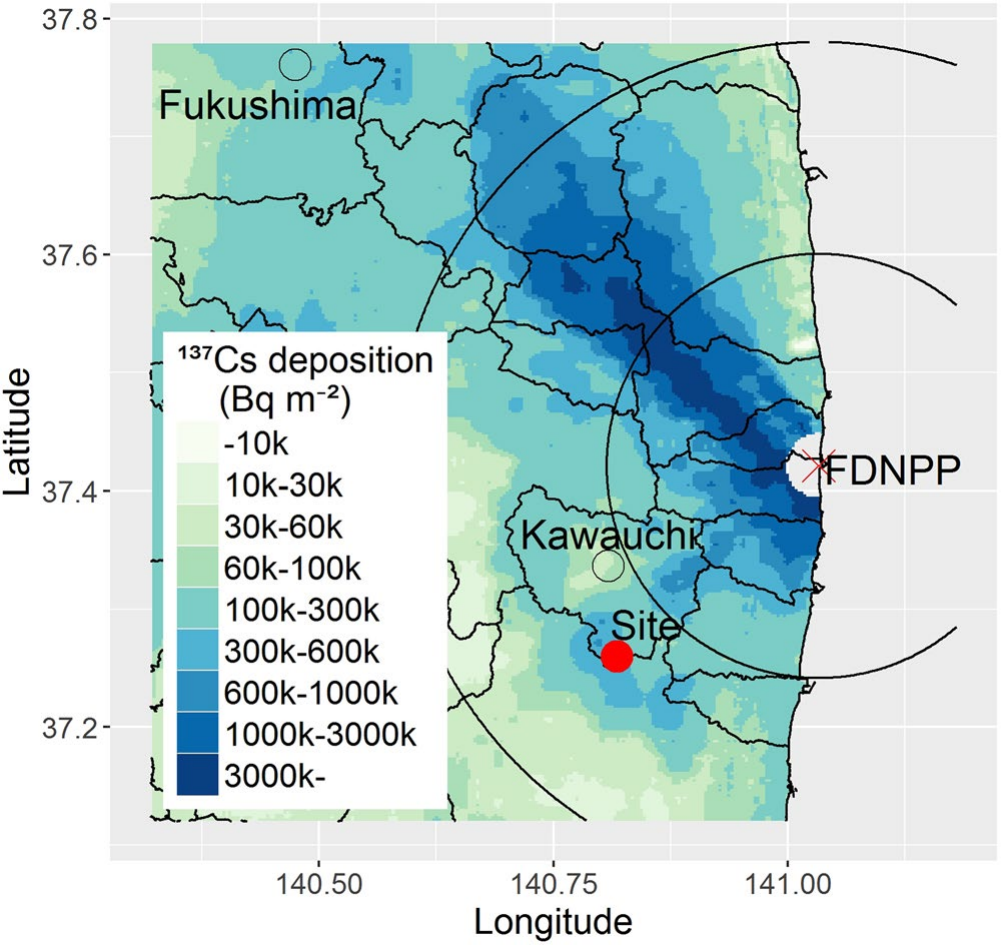
The location of the study site. The study site is located at the south side of Kawauchi village and 26 km away from the FDNPP. The two circles indicate 20 and 40 km from the FDNPP, respectively. The ^137^Cs deposition data were obtained from the airborne monitoring survey by prefectures (decay corrected on 31 May 2012)^36^. The municipal boundaries were generated from Administrative Zones Data^37^. The map was created using R version 3.4.0^31^ and ggplot2 package^38^.

In this study, to understand the behaviour of radiocesium among the afforested areas and develop the technique for decreasing radiocesium transfer to trees, a mixed forest of pine and deciduous broad-leaved trees in the Kawauchi village 26 km apart from the FDNPP was clearly cut, and the seedlings of Japanese cypress (*Chamaecyparis obtusa*), which is one of the major conifer species, were planted (Fig. 1). Japanese cypress prefers temperate climate, and it is largely planted (one-third of Japanese cedar (*Cryptomeria japonica*), the most dominant tree species, as area) in the relatively warm coastal area surrounding the FDNPP, while it is rarely cultivated on the inland of Fukushima prefecture^17^. The research site has infertile soils and it is adequate to Japanese cypress than cedar.

**Figure 2:**
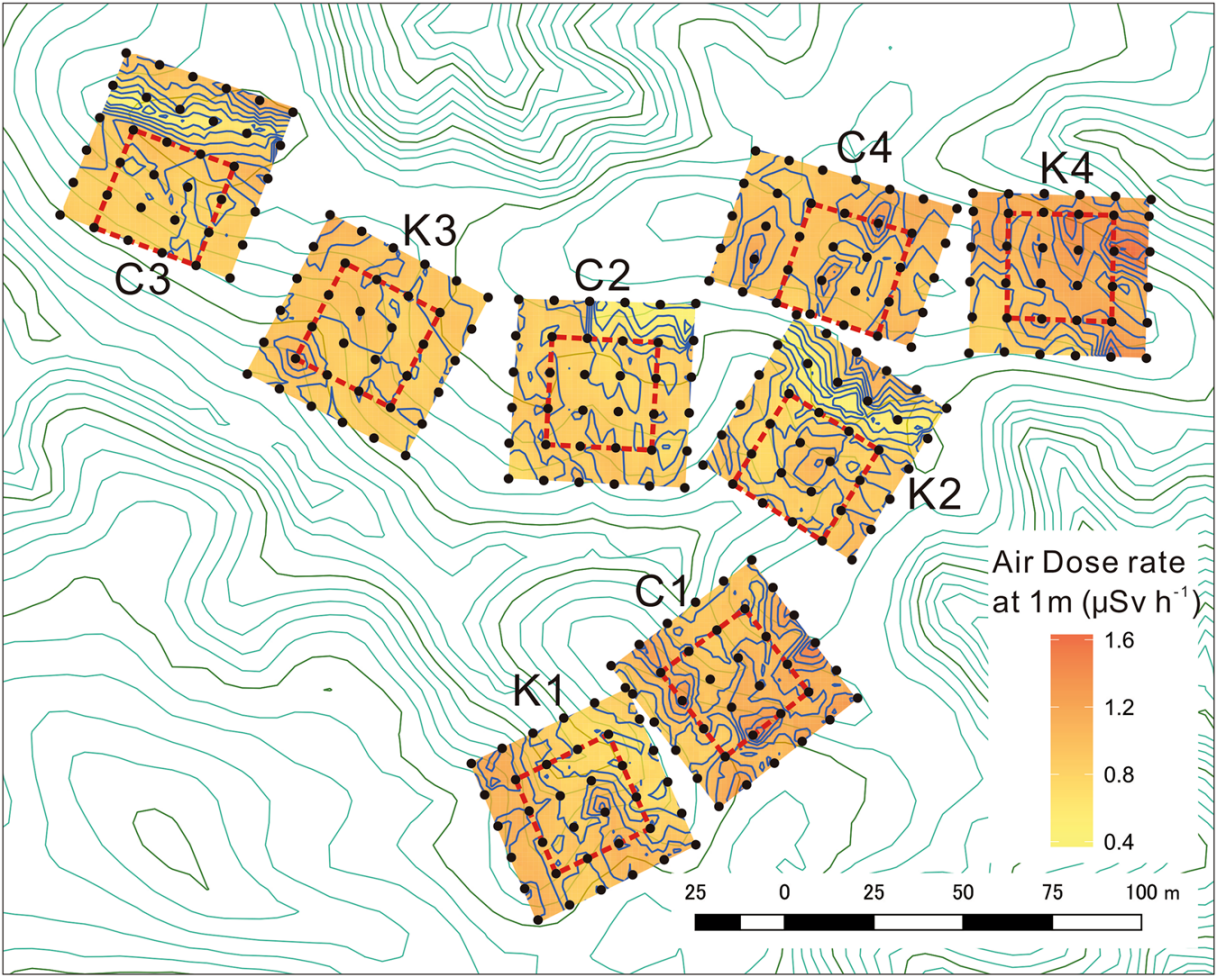
The map of the study site. Eight plots involving four K-fertilised plots (K1–4) and four control plots (C1–4) were established. Plot size was 50 × 50 m, except for K4 (45 × 50 m) and C4 (40 × 60 m). The spatial distribution of air dose rate at 1-m height was visualised by contour lines (blue colour, 0.1 µSv h^−1^ intervals) and colour gradient within plots (measured in Augst 2014). Black points: 10-m grid within plots, Red dot lines: boundaries of 30 × 30 m quadrat for sampling, Dark green lines: contour lines indicating 10-m elevation, Light green lines: contour lines indicating 2-m elevation. The contour lines of elevation were generated from 5-m-resolution DEM^39^.

To elucidate the prolonged effects of K fertilisation on the uptake of soil radiocesium, eight plots measuring 0.225–0.25 hectare (ha) were established in the site (Fig. 2) and 83 kg K ha^−1^ was applied on half of the plots twice in August 2014 and April 2015 (total 166 kg K ha^−1^). In each plot, five seedlings and nearby soils were sampled, and the random effect of the plot was considered in the analysis. We demonstrate and discuss the results of the first 2 years of K fertilisation experiment conducted in a stand contaminated by the FDNPP accident and planted with *C. obtusa* seedlings without initial radiocesium contamination.

## Results

### Physicochemical properties of soils

The physicochemical properties of surface (0–5 cm) and deeper (5–10 and 10–20 cm) soil layers just before fertilisation in August 2014 and the surface soil layers in October 2015 were recorded (Table 1). No significant differences in the soil physicochemical properties were observed between the C- and K-plots (nested ANOVA, p > 0.05). In addition, the exchangeable K concentrations in surface soils in October 2015 (after fertilisation) did not differ from those obtained in August 2014 (before fertilisation). A linear mixed model (LMM) analysis was conducted for August 2014 samples using the soil exchangeable K concentrations as a response variable, the weight of organic layer as an explanatory variable and the sampling plot as a random effect. In the analysis, the regression coefficient was positively significant (p = 0.009).

**Table 1:** Physicochemical properties of soils.

Date	Soil layer	Treatment	C	N	pH(H_2_0)	pH (KCl)	CEC	Ex. Ca	Ex. Mg	Ex. K	Ex. Na	Ex. Cs
			[g kg^−1^]				[cmol(+) kg^−1^]					[µmol(+) kg^−1^]
2014/8	0–5 cm	K-fertilised	182 ± 41	8.17 ± 1.41	4.24 ± 0.17	3.38 ± 0.26	50.8 ± 11.5	3.26 ± 1.67	1.23 ± 0.62	0.820 ± 0.155	0.15 ± 0.05	0.292 ± 0.140
		Control	152 ± 12	6.92 ± 0.88	4.34 ± 0.09	3.48 ± 0.07	38.7 ± 8.3	1.39 ± 0.85	0.65 ± 0.19	0.614 ± 0.109	0.14 ± 0.04	0.344 ± 0.075
	5–10 cm	K-fertilised	95 ± 46	4.30 ± 1.53	4.78 ± 0.22	3.90 ± 0.29	27.5 ± 11.9	1.23 ± 1.92	0.28 ± 0.17	0.325 ± 0.150	0.08 ± 0.02	NA
		Control	122 ± 40	5.23 ± 1.43	4.60 ± 0.35	3.83 ± 0.26	32.8 ± 12.7	0.35 ± 0.17	0.28 ± 0.05	0.425 ± 0.150	0.10 ± 0.07	NA
	10–20 cm	K-fertilised	64 ± 29	2.83 ± 0.71	4.90 ± 0.08	4.18 ± 0.13	16.7 ± 5.3	0.30 ± 0.27	0.12 ± 0.05	0.125 ± 0.050	0.06 ± 0.01	NA
		Control	76 ± 30	3.23 ± 1.10	4.85 ± 0.17	4.15 ± 0.17	18.9 ± 7.3	0.25 ± 0.13	0.15 ± 0.06	0.168 ± 0.104	0.07 ± 0.02	NA
2015/10	0–5 cm	K-fertilised	173 ± 52	7.36 ± 1.20	3.85 ± 0.27	3.73 ± 0.29	49.2 ± 12.1	2.81 ± 1.45	1.23 ± 0.55	0.816 ± 0.344	0.18 ± 0.04	0.323 ± 0.155
		Control	172 ± 41	7.54 ± 2.36	3.87 ± 0.10	3.56 ± 0.19	51.1 ± 10.4	4.32 ± 3.11	1.68 ± 1.08	0.618 ± 0.069	0.22 ± 0.09	0.302 ± 0.109

For surface soil layer (0–5 cm), 40 samples (five replicates for each plot) were used, and for deeper soil layers (5–10 and 10–20 cm), 16 samples (one replicate for each plot) were used. In the analysed properties, C, N, pH(H_2_O) and pH(KCl) of some samples were not analysed because of small sample amount. The values were shown as mean ± standard deviation. NA: not analysed.

### ^137^Cs concentrations in seedlings

**Figure 3:**
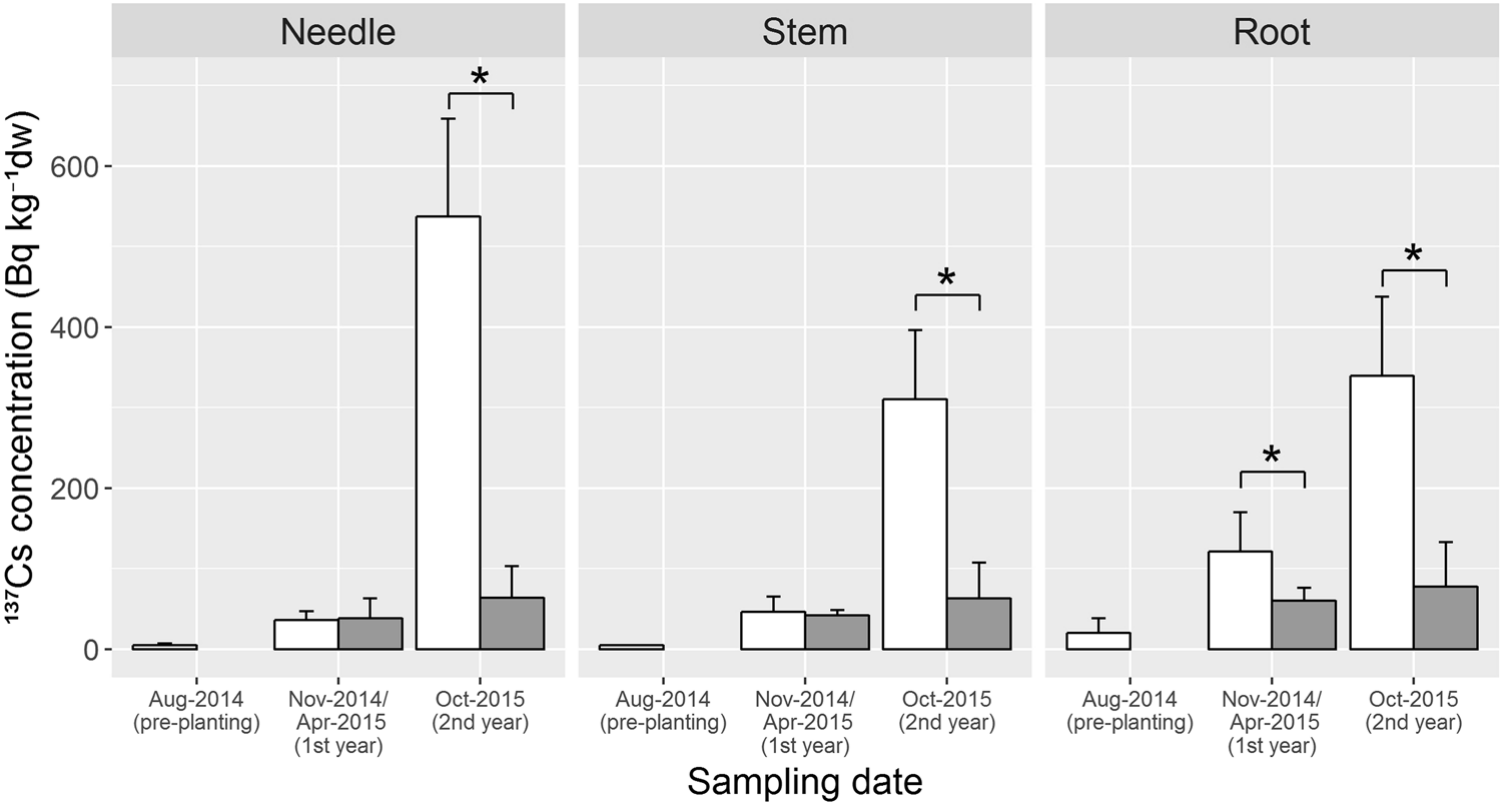
^137^Cs concentrations in the cypress seedlings sampled at control plots (open bar) and K-fertilised plots (grey bar). Asterisks (*) indicate that samples obtained at the same time showed significant differences between treatments (nested ANOVA, p < 0.05). All stem samples at pre-planting showed less values than the detection limit despite long (24-h) measurements. Subsequently, the mean of detection limits was shown.

**Figure 4:**
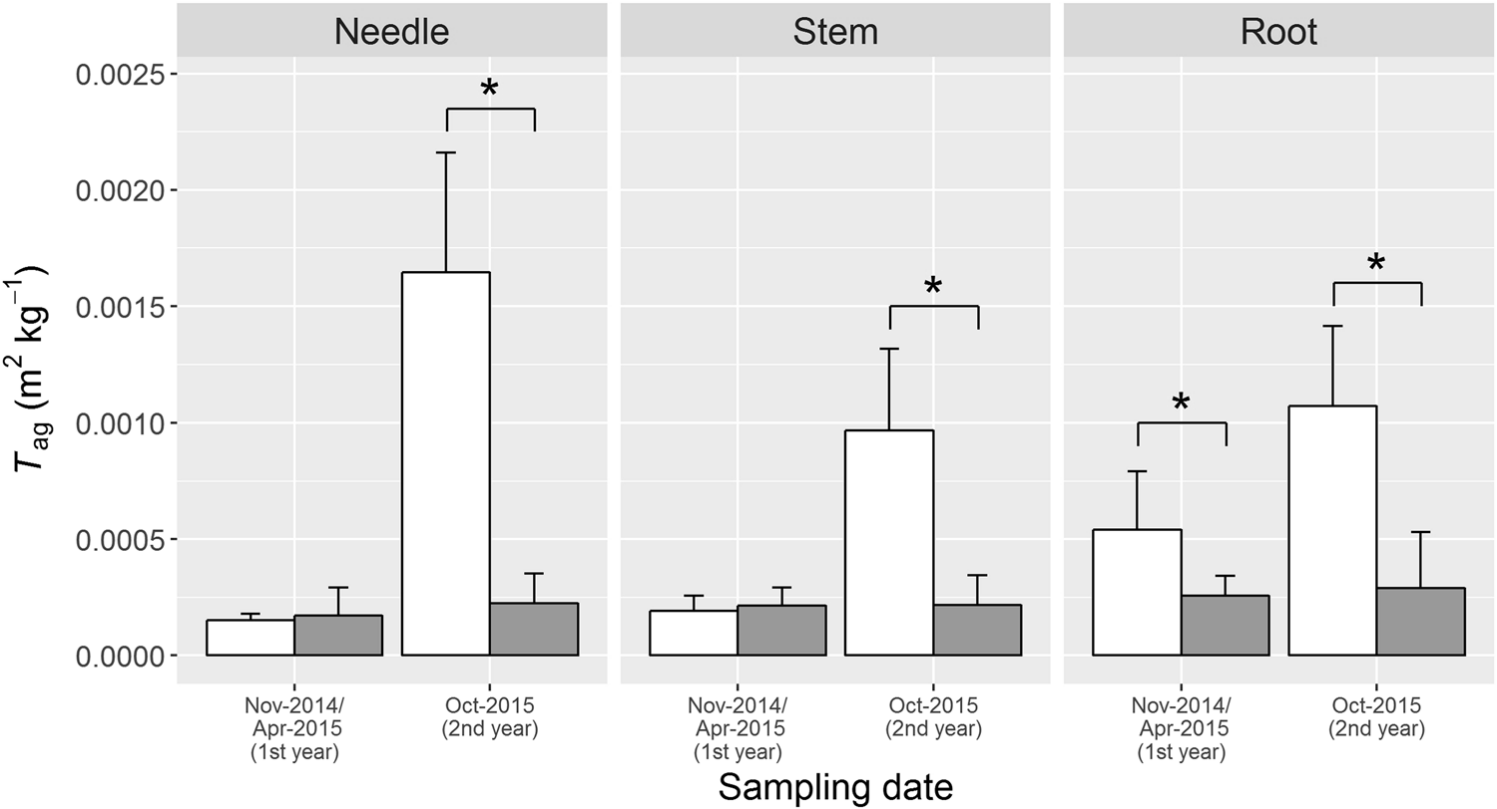
Aggregated transfer factor (*T*_ag_) of cypress seedlings sampled at control plots (open bar) and K-fertilised plots (grey bar). Asterisks (*) indicate that samples obtained at the same time showed significant differences between the treatments (nested ANOVA, p < 0.05).

Initial ^137^Cs concentrations in the Japanese cypress (*C. obtusa*) seedlings (pre-planting) were 4.81 ± 2.44 Bq kg^−1^ in needles, <4.89 Bq kg^−1^ in stems (all samples had less than the detection limit) and 20.6 ± 17.6 Bq kg^−1^ in roots (Fig. 3). The ^137^Cs concentrations in the seedlings harvested after the first growing season (November 2014 and April 2015, 1st year) were higher than those in the pre-planted seedlings (August 2014), and the roots sampled from control (C-) plots had significantly higher ^137^Cs concentrations than those from the K-fertilised (K-) plots (nested analysis of variance (ANOVA), p < 0.05). On the other hand, there were no significant differences between treatments in the needles and stems after the first growing season. ^137^Cs concentrations in the seedlings sampled in October 2015 (2nd year) were significantly different between the C- and K-plots (Fig. 3), wherein the ^137^Cs concentrations in the seedlings from C-plots were higher (300–550 Bq kg^−1^ in average) than those in the seedlings sampled in the previous year (<100 Bq kg^−1^ in average), whereas the increment in K-plots was less evident. The *T*_ag_ value showed a similar trend as that of ^137^Cs concentrations (Fig. 4). The values after the first growing season or the values in the K-plots were usually <0.0025 m^2^ kg^−1^, whereas the seedlings from the C-plots showed *T*_ag_ > 0.01 m^2^ kg^−1^ after the second growing season. ^137^Cs concentrations in each compartment of the seedlings increased with increasing ^137^Cs inventory of soil and were decreased by K treatment (Supplementary Fig. S1).

**Figure 5:**
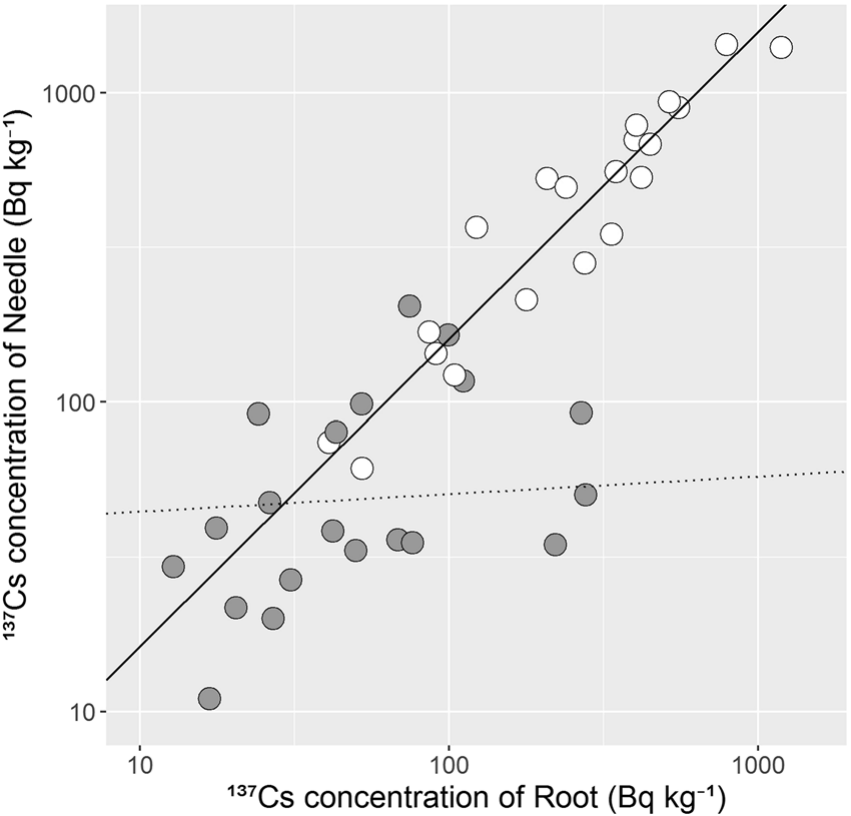
Relationship between ^137^Cs concentrations among compartments (needles and roots) in control (open symbols) and K-fertilised (grey symbols). Both axes are shown by logarithmic scales. The seedlings were sampled in October 2015 at the end of the second growing season. Two linear regression lines were drawn for control (solid line) and K-fertilised (dotted line) because a significant interaction between logarithmic root ^137^Cs concentration and fertilisation treatment was observed in the regression analysis (nested ANCOVA, p < 0.05).

^137^Cs concentrations in the needles and roots sampled in October 2015 showed positive correlations (Fig. 5). However, there was an interaction between root ^137^Cs concentration and fertilisation (nested analysis of covariance (ANCOVA), p < 0.05), and the slope parameter of root ^137^Cs concentration decreased in the K-plots compared to that in the C-plots. The ratio of ^137^Cs activity of needles to that of roots was 1.66 ± 0.51 in C-plots and 1.29 ± 0.96 in K-plots (n = 20).

The online Supplementary Table S1 shows the height, the diameter of the ground surface and the weight of the compartments. The average height of the seedlings was about 50 cm while planting in August 2014 and increased by about 2 cm in November 2014, the end of the growing season. The tree height increased to more than 70 cm in average in the next autumn. The needles, stems and roots of the seedlings sampled for radioactive analysis in August 2014 weighed 14.3 ± 4.2, 11.2 ± 3.2 and 10.6 ± 3.2 g, respectively (mean ± SD, n = 20). Thereafter, the needle weights decreased and the root weights increased in the first autumn. The sample weights of all compartments increased in October 2015 as follows: 36–41 g for needles, 30–32 g for stems and 18 g for roots. No differences in both tree heights and sample weights were observed among the plot treatments.

The online Supplementary Table S2 shows the nutrient concentrations of cypress needles sampled for measurement of radiocesium concentration in autumn 2015. The needle potassium concentration in K-plots was significantly higher than that in C-plots, but, to the contrary, the concentrations of magnesium, calcium and manganese were significantly lower in K-plots than those in C-plots (nested ANOVA, p < 0.05).

### Relationship between ^137^Cs activity of seedlings and soil properties

**Figure 6:**
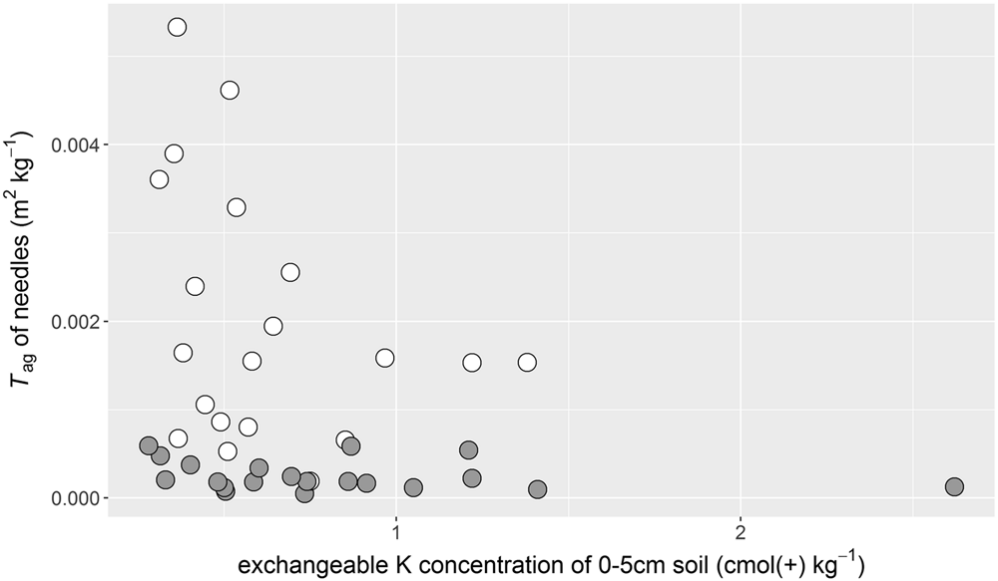
Relationship between exchangeable K concentrations of surface soil (0–5 cm) and aggregated transfer factor (*T*_ag_) of cypress needles in control (open symbols) and K-fertilised (grey symbols). The seedlings were sampled in October 2015 at the end of the second growing season.

The relationship between *T*_ag_ of needles and surface soil concentrations of exchangeable K is shown in Fig. 6. In the C-plots, some seedlings with exchangeable K concentrations < 0.50 cmol (+) kg^−1^ had *T*_ag_ values > 0.002 m^2^ kg^−1^. The *T*_ag_ values of all seedlings in K-plots were < 0.001 m^2^ kg^−1^ and obviously smaller than those of the seedlings in C-plots regardless of the soil exchangeable K concentration.

The results of a LMM analysis for cypress needles sampled in autumn 2015 are shown in Table 2. The variation in ^137^Cs concentrations in the needles (*Cs*_needle_) can be explained by the following equation:

2 $$\mathrm{log}\,C{s}_{{\rm{needle}}}=-6.94+1.01\,\mathrm{log}\,C{s}_{soil\_inv}-1.90K-0.54\,\mathrm{log}\,exK+re$$

where logarithmic soil ^137^Cs inventory (*Cs*_soil_inv_), K fertilisation (*K*) and logarithmic exchangeable K concentrations in surface soil (*exK*) are significant variables for fixed effects (p < 0.05). The variable of random effect of sampling plots (*re*) was 0.11. In general, since soil exchangeable K concentrations would be directly affected by K fertilisation, using both exchangeable K and fertilisation as explanatory variables is believed to be not preferable. However, the soil exchangeable K concentration was not changed before and after fertilisation (Table 2). Therefore, we used both factors in the analysis, in which effects of the *exK* in the equations were likely to result from spatial variation of soil exchangeable K concentrations (Fig. 6). The Akaike’s Information Criterion (AIC) of the above model shown by equation (2) was 96.4. The model using logarithmic cation exchange capacity (*CEC*) as the explanatory variable instead of soil ^137^Cs inventory in the previous model (equation (2)) showed the minimum AIC (AIC = 90.9).

3 $$\mathrm{log}\,C{s}_{{\rm{needle}}}=-2.82+2.10\,\mathrm{log}\,CEC-1.77K-0.94\,\mathrm{log}\,exK+re$$

**Table 2:** Parameter estimates, SE, t and p values of fixed effect; variance and SD of random effect for logarithmic ^137^Cs concentrations of Japanese cypress needles sampled in October 2015 using linear mixed model.

		Estimated coefficients	SE	t value	p value
Model1	Fixed effect				
AIC: 96.4	(Intercept)	−6.95	3.97		
	log(soil ^137^Cs inventory)	1.01	0.32	3.20	0.003
	K fertilisation	−1.90	0.32	−6.07	0.0003
	l og(soil ex K)	−0.54	0.24	−2.29	0.028
	Random effect	Variance	SD		
	Plot	0.11	0.33		
Model2	Fixed effect				
AIC: 90.5	(Intercept)	−2.82	1.99		
	log(CEC)	2.11	0.49	4.32	0.0002
	K fertilisation	−1.77	0.27	−6.68	0.0002
	log(soil ex K)	−0.94	0.25	−3.74	0.0006
	Random effect	Variance	SD		
	Plot	0.06	0.24		

These models were performed with Gaussian distribution and identity link function: soil ex K; soil exchangeable K concentrations of surface (0–5 cm) soil, CEC; cation change capacity.

Although the carbon contents positively correlated with CEC (R = 0.89, p < 0.001), the carbon contents were not used in the LMM analysis because of lack of analysis due to small sample amount (n = 25). The models involving other factors such as other soil physicochemical properties, size of the sampled seedlings and slope angle of the sampling site showed no or slight decrease (*Δ*AIC < 0.3) compared with the former two models.

## Discussion

This study aimed to elucidate the effects of K fertilisation on radiocesium transfer from soil to trees planted after the FDNPP accident in 2011. We planted Japanese cypress seedlings in a clear-cut forest in Fukushima prefecture in early summer 2014, and K fertiliser was applied twice (in August 2014 and in April 2015) (166 kg ha^−1^ K in total) in the K-fertilised plots. Seedlings harvested after the first growing season (autumn 2014 and spring 2015) did not show a difference in ^137^Cs concentrations between K-fertilised and control plots (K- and C- plots, respectively), except in roots. During the second growing season, however, ^137^Cs concentrations in the seedlings from the C-plots were much increased than these from the K-plots. Consequently, ^137^Cs concentrations in the seedlings from the K-plots were one-eighth of those in the seedlings from the C-plots (Fig. 3). The *T*_ag_ value also showed similar patterns of variation in ^137^Cs concentrations in the plant samples (Fig. 4). After the FDNPP accident, the suppressive effects of K fertilisation on the uptake of radiocesium by crops have been reported by several papers^13,14,18,19^, but, to our knowledge, no study has been conducted for field-grown tree species. Thus, this is the first study clearly showing the suppressive effects of K fertilisation on radiocesium uptake by trees growing on the soil contaminated by the FDNPP accident. In the first growing season, the root slightly grew probably for root-taking, whereas there was no growth in the needles and stems (Supplementary Table S1). At the same time, there was no difference in ^137^Cs concentrations in the needles and stems between the K- and C-plots. This is probably because the uptake of radiocesium occurred with growth.

Several previous studies have suggested that K fertilisation suppressed radiocesium absorption by pine trees^15^, shrubs and mushrooms^20^ in mature stands contaminated by the ChNPP accident. Those results showed that ^137^Cs concentrations in plants and fungi in K-fertilised sites decreased by a maximum of ca. 60% compared with those in unfertilised sites. Our experiments, on the other hand, demonstrated higher suppressive effects of K fertilisation (by 88% decrease in Cs uptake) than those reported previously. This is probably because we fertilised small seedlings intensively in a short period (i.e. twice with 83 kg ha^−1^ of K in 2 years) immediately after the planting, whereas the previous studies investigated mature stands that were fertilised only once after the accident or fertilised several times, but less intensively, since 1960s. Those studies also demonstrated that the effects of fertilisations, including those before the accident, lasted for more than 10 years, although the K fertilisations after the accident reduced the ^137^Cs levels in the plants more obviously. In contrast, radiocesium deposited on the crowns of standing trees was immediately taken up via the surface of leaves and/or branches at the initial stage of the deposition (direct contamination)^7,9^ and would remain in the trees for a long time^10^. Taken together, the results of our study suggested that K fertilisation can be more effective to newly planted seedlings than to larger trees that received the direct above-ground deposition for decreasing the levels of radiocesium contamination.

For seedling samples harvested in autumn 2015, a positive correlation was observed in ^137^Cs concentrations between needles and roots (Fig. 5). These results showed that K fertilisation inhibited root uptake of radiocesium and consequently suppressed the increase in needle ^137^Cs concentrations. K fertilisation has been shown to affect not only root uptake of radiocesium but also translocation to other plant parts in rice^18^. In our analysis, the slope was significantly lower in K-plots than that in C-plots, suggesting that the fertilisation might also inhibit radiocesium translocation from the root to needles of cypress seedlings.

In autumn 2015, the physicochemical properties of surface mineral soils, including exchangeable K concentrations, were surveyed, and the relationship between *T*_ag_ values and exchangeable K concentrations seemed to show an inverse variation in C-plots, and all samples with *T*_ag_ values >0.002 m^2^ kg^−1^ in the needles of the C-plots were from locations with soil exchangeable K concentrations <0.50 cmol (+) kg^−1^. The relationships between the soil–plant radiocesium transfer and soil exchangeable K concentrations were also studied in a paddy field contaminated by the FDNPP accident. These studies suggested that the threshold level of soil exchangeable K concentrations was 25 mg K_2_O per 100 g of soil for reducing the radiocesium uptake by rice^14,21^. This value is equal to 0.53 cmol (+) K kg^−1^, which was similar to the threshold soil K concentration observed in our cypress stand (Fig. 6), despite the large differences in soil conditions between the forest soil and submerged paddy soil. Moreover, our results clearly showed that K fertilisation effectively reduced the radiocesium transfer in the case of such a low exchangeable K condition.

We initially hypothesised that increased soil exchangeable K concentrations due to fertilisation would suppress radiocesium root uptake by cypress seedlings in the fertilised plots. The soil exchangeable K concentrations, however, were unchanged by fertilisation, in contrast to our expectations (Table 1). Moreover, *T*_ag_ values of needles in the K-plots were much lower than those of needles in the C-plots even when the soil exchangeable K concentrations were <0.50 cmol (+) kg^−1^ (Table 2 and Fig. 6). Since the analysed soil samples were collected at the end of the growing season in this study, these low levels of soil exchangeable K concentrations in the fertilised plots probably resulted from K uptake by the cypress seedlings in the growing season, as has been reported for peach trees that absorbed and accumulated K in summer^22^. Actually, because the fertilised seedlings showed increased K concentrations in the needles (Supplementary Table S2), it was concluded that these seedlings absorbed fertilised K from the soil. Moreover, the soluble K can easily penetrate into deeper layers of the soil, which would decrease the K concentrations in the surface soil^23^. Therefore, it was suggested that the levels of exchangeable K would temporarily increase by fertilisation during the growing season, and radiocesium uptake by tree roots was suppressed. An LMM analysis indicated that the soil exchangeable K concentrations in the surface soils linearly increased with the increasing amount of organic layer in August 2014, just after the planting. Since tree composition and the rate of litter decomposition are expected to be constant among the plots, the amount of organic layer amount might reflect the ability of K supply. In future studies, it would be better to examine exchangeable K concentrations in the soil at the beginning of the growing season after the fertilisation for analysing the relationships between soil–plant radiocesium transfer and exchangeable K concentrations in the soil.

Another LMM analysis for estimating the needle ^137^Cs concentration indicated that the coefficients of soil ^137^Cs inventories, fertilisation treatment and surface soil exchangeable K concentrations were significant (Table 2). These results suggested that such parameters could be factors determining the amount of radiocesium absorption. In particular, in the model, the coefficient of logarithmic ^137^Cs inventories was nearly one (equation (3)). This result indicated that the relationship of radiocesium between needles and soils was linear, but not exponential. If the coefficient of ^137^Cs inventories in equation (2) was fixed as one, the equation was transformed to the equation of *T*_ag_ as follows:

4 $$\mathrm{log}C{s}_{{\rm{needle}}}/C{s}_{soil\_inv}=\,\mathrm{log}\,{T}_{{\rm{ag}}}=-6.94-1.90K-0.54\,\mathrm{log}\,exK+re$$

This equation shows that the *T*_ag_ values were affected by soil exchangeable K and fertilisation as visualised in Fig. 6.

On the other hand, the model that used CEC as an explanatory variable instead of ^137^Cs inventories was selected as the best model because of its minimum AIC (Table 2). Although it is difficult to conclude the mechanism from these results, since CEC showed a positive correlation with the soil carbon content, it was possible that the soil anion site derived from the organic matter might hold radiocesium cation and supply it to trees. Such a hypothesis coincided with the results that organic anion has weak adhesion capacity of cesium and suppresses the cesium adhesion to clay minerals^24^. Further research resolving the factor of radiocesium transfer from soils to trees is necessary to promote the suppression of radiocesium transfer.

We demonstrated that K fertilisation could suppress the radiocesium root uptake by planted cypress seedlings in a forest highly contaminated with radiocesium due to the FDNPP accident. Previous reports had indicated that the suppressive effects of K fertilisation on the soil–plant radiocesium transfer varied with the amounts and frequency of fertilisation and the soil conditions receiving the fertilisation^15,25^. On the other hand, we may need to monitor the adverse effects of fertilisation on tree growth. The fertilised needles showed significantly decreased Mg, Ca and Mn concentrations than those of unfertilised needles, but their K concentrations were increased. These results clearly indicated that K fertilisation altered the nutritional balance of seedlings. In our cypress stand, the total amount of exchangeable K concentrations in mineral soil (0–20 cm depth) was 10.4 g m^−2^, which is comparable to the amount of fertilisation (8.3 g m^−2^) in this study. Although the amount of fertilisation appears not to be excessive, repeated fertilisation may cause lack of other cations in the soil or nutritional imbalances and consequently may exert harmful effects on tree growth. Therefore, an important issue for future studies would be providing information on amounts and frequency of the K fertilisation that can be effective for long periods needed for wood production without adverse effects on tree growth. We expect that long-term monitoring of the cypress stand used in this study will deepen our insights into soil–tree radiocesium transfer, which in turn, would help to establish practical techniques for wood production in the area with radiocesium deposition.

## Materials and Methods

### Site

This study was conducted in Mariyama area of Kawauchi village in Fukushima prefecture (37°16′ N, 140°49′ E, Fig. 1), which is 26 km away to the southwest from the FDNPP. This study site belongs to the Abukuma mountainous area, and its altitude is 680 m, where the mean annual temperature and precipitation were 10.3 °C and 1465 mm (data obtained at Kawauchi monitoring station of AMeDAS, Japan Meteorological Agency, 37°20′ N, 140°49′ E), respectively. The study site has a complex micro-topographical conditions, including a stream and a ridge, where slopes range from 0 to 36 degrees with varied aspects (Fig. 2). The ^137^Cs deposition in the studied area was estimated to be 380–400 kBq m^−2^ by the fourth airborne monitoring conducted from October 25 to November 5 in 2011^26^.

The study site had been a mixed forest of Japanese red pine (*Pinus densiflora*) and deciduous broad-leaved trees such as oak (*Quercus serrata* and *Q. mongolica* var. *crispula*). The soil is classified as brown forest soil^27^, in which the parent material is granite affected by volcanic ash on the surface. The soil texture is light clay (LiC) containing 280 g kg^−1^ of clay, and vermiculite is dominant with clay minerals. These mineral soil properties are common in the Abukuma mountainous area. The trees in the 4-ha study site were clear-cut during winter to spring in 2014, and all the logs and branches were stacked in the site along the contour.

Three-year-old seedlings of Japanese cypress (*C. obtusa*), which were grown at a nursery in Minami-Soma (ca. 44 km north east from the study site), were planted at a density of about 2,800 plants per hectare during June to July in 2014. Before planting, 20 seedlings were sampled and their heights, diameters at the ground surface and weights of the needles, stems and roots were measured. Moreover, the initial radiocesium levels were determined in 5 of the 20 seedlings. The dwarf bamboo growing on the ground surface was periodically mowed.

### Plot set-up, fertilisation and monitoring

In July 2014, eight plots were established (Fig. 2) in the study site, six of them were 0.25 ha (50 × 50 m) and the other two plots were 0.225 ha (45 × 50 m) and 0.24 ha (40 × 60 m), respectively, due to the location of the work road and the micro-topographical conditions of the stand. Furthermore, a 0.09-ha quadrat (30 × 30 m) was set up in each plot for sampling for radionuclide analysis. Among those eight plots, four were applied with K fertiliser, and the other four plots were used as unfertilised control plots (Fig. 2). In November 2014 and October 2015, the heights and diameters at the ground surface of seedlings growing in the eight quadrats were measured.

K fertilisation was conducted in August 2014 and April 2015, in each of which potassium chloride fertiliser (60% KCl) was applied at the rate of 83 kg K per hectare (ha). Thus, the total amount of K fertilisation was 166 kg K ha^−1^.

In August 2014, the air dose rate at 1-m and 5-cm height was measured above the 10-m grids in all the eight plots (50 × 50 m, etc.) using dual plastic scintillators equipped with a GPS system (Gamma plotter H, Japan Atomic Energy Agency).

### Samplings

Accumulated organic matters on the ground and mineral soils were sampled at five points in each quadrat just before the first fertilisation in August 2014. To collect the organic matter samples, a square frame with an area of 0.625 m^2^ (25 × 25 cm) was set on the ground at each sampling location, and the organic matters in the frame were manually collected^28^. In one of those five sampling locations, mineral soil samples were collected using a 475-mL core sampler (φ11 cm, 5 cm height, DIK-1815-11, Daiki Rika Kogyo Co. Ltd., Tokyo, Japan) from the soil layers at 0–5, 5–10, 10–15 and 15–20 cm depths^28^. In the remaining four sampling locations, mineral soils were manually sampled using small shovels at 0–5, 5–10 and 10–20 cm depth layers.

In November 2014, a seedling was harvested in each quadrat by pulling up a whole plant, including the roots, using a shovel. At the same time, organic matter and mineral soil samples close to the seedling (within 50 cm) were also collected. In April 2015, just before the second fertilisation, four sets of the samples were additionally collected in each quadrat. These five sets of samples (seedlings, organic matter and mineral soil) were considered as samples after one growing season in the K fertilisation experiment, because April is the end of the dormant season (before the second growing season) in this study site. In those samplings, organic matter was collected in the same way as initial sampling in August 2014, whereas soil samples below the organic layers were collected as soil cores with 0–20 cm soil depth and 5-cm diameter using a soil auger (HS-25S, Fujiwara Scientific Company, Tokyo, Japan). The 20-cm soil cores were divided into four pieces with 5-cm depth intervals in the laboratory.

In October 2015, five seedlings and nearby soils were sampled in each quadrat. The sampling was carried out in the same way as done in November 2014 and April 2015.

### Sample preparation and analyses

The plant and soil samples were transported to the laboratory and subjected to the following sample preparation and measurements. The seedlings were divided into needles, stems (with branches) and roots using scissors and were washed with tap water and subsequently with deionised water. The washed needles, stems, roots and the accumulated organic matters were dried at 75 °C for > 48 h and weighed. Thereafter, the dried samples were milled and packed into 100-mL plastic containers (U8 case) for measuring the radioactivity. The mineral soils were air-dried at room temperature for more than 2 weeks. These soil core samples were weighed to determine the bulk density of the soil. The bulk densities of the soil core samples collected in August 2014 were used as substitutes for those of manually sampled soils in the same plots. The air-dried soil samples were sieved through a 2-mm mesh, and the fine soils were packed into the 100-mL plastic containers (U8 case).

The sample activity of ^134^Cs and ^137^Cs (Bq kg^−1^) was measured using an HPGe coaxial detector system (GEM20-70, Seiko EG&G, Tokyo, Japan) at FFPRI. The measurement time was 3600–86400 s until the standard errors became <10% of the measured activity. If the measurement activity was below the detection limit despite measurement for a long time, the detection limit was used for statistical analysis as described later. The measurement of radioactivity has been described in detail by Komatsu *et al*. (2016)^3^ and Kuroda *et al*. (2013)^29^. The radiocesium activity of some samples was measured at the Kankyo Research Co. Ltd. (Tokyo, Japan). The ^137^Cs inventories (Bq m^−2^) in the organic layer and the mineral soil layers were calculated using ^137^Cs concentrations (Bq kg^−1^) and dry masses of organic layers per unit area or bulk density of the mineral soils (kg m^−2^). *T*_ag_ of each compartment was calculated from equation (1).

Elemental analysis was conducted for the needles sampled for radiocesium analysis in the second growing season. The concentrations of Na, Mg, Al, P, K, Ca, Mn, Fe and Zn in the needle samples were measured after wet digestion by HNO_3_ and H_2_O_2_ (4:1) using ICP-MS (Agilent 7700x, Agilent Technologies). Among the mineral soil samples, the physicochemical properties of 40 (five replicates in one quadrat) soil samples at the surface layer (0–5 cm) and 16 (one replicate) soils in the deeper layers (5–10 and 10–20 cm) in August 2014 and 40 surface soils in October 2015 were analysed. For the physicochemical analyses, sieved fine soils were used, and core samples of 10–15 and 15–20 cm were mixed. Soil pH (H_2_O) was measured by a glass-electrode method, in which the soil:liquid ratio was 1:2.5. Exchangeable cations (Ca, Mg, K, Na and ^133^Cs) in the soil samples were extracted with 1 mol L^−1^ ammonium acetate solution at pH 7. The concentrations of exchangeable cations were determined using ICP-MS (Thermo Fisher Scientific iCap Q). Cation exchange capacity (CEC) was analysed according to Schollenberger method^30^, in which the soil samples were shaken with 1 mol L^−1^ ammonium acetate solution at pH 7 and then with 10% NaCl; following Kjeldahl distillation, the ammonium ion content of the solution was then determined by titration with NaOH. Total carbon and nitrogen levels in the soils were analysed by dry combustion method.

### Statistical analysis

Samples were collected by nested design (five replicates from eight plots). To assess the factors affecting the log-transformed ^137^Cs concentrations in the seedlings sampled in autumn 2015, an LMM analysis was performed involving sampling plots serving as a random effect. In this analysis, plot treatment (with fertiliser or not), log-transformed total soil ^137^Cs inventory, physicochemical properties of 0–5 cm soil, plant size parameters (height, diameter and weights) and slope angle of the sampling site were used as candidate explanatory variables. The best model was selected based on stepwise AIC method. In the LMM analysis, maximum likelihood estimation was used for estimating model parameters. For evaluating the effect of fertiliser on the soil physicochemical properties and/or radiocesium concentrations in the seedlings sampled at the same time, a nested ANOVA was conducted with sampling plots serving as a random effect. These statistical analyses were performed by R version 3.4.0^31^, and LMM analysis was done by R packages of lme4^32^ and lmerTest^33^. The mean and standard deviation values, including the censored data, were obtained by maximum likelihood estimation^34^ using R packages of NADA^35^.

## Electronic supplementary material

Supplementary Figure S1 and Table S1 and S2
