# Supplementary material for: Potassium fertilisation reduces radiocesium uptake by Japanese cypress seedlings grown in a stand contaminated by the Fukushima Daiichi nuclear accident
Source: Sci Rep. 2017 Nov 15;7:15612. doi: 10.1038/s41598-017-15401-w (PMC5688087; doi:10.1038/s41598-017-15401-w)
Supplement: Supplementary file 1 — Supplementary Figure S1 and Table S1 and S2 [file 41598_2017_15401_MOESM1_ESM.pdf]

Potassium fertilisation reduces radiocesium uptake by Japanese cypress seedlings grown in a stand contaminated by the Fukushima Daiichi nuclear accident

Masabumi Komatsu, Keizo Hirai, Junko Nagakura, Kyotaro Noguchi

Supplementary Table S1. Height, diameter at ground surface and weight of compartment of Japanese cypress seedlings.

Date	Treatment	Height [cm]	Diameter on ground surface[mm]	Weight[g]		
				Needle	Stem	Root
2014/8	K-fertilised	47.4± 4.2	6.6±0.8	ND	ND	ND
	Control	50.8± 2.2	6.9±0.1	14.3±4.2	11.2±3.2	10.6±3.1
2014/11, 2015/4	K-fertilised	49.4± 5.0	7.2±0.8	10.6±2.9	12.8±2.6	14.8±1.9
	Control	52.9± 1.7	7.8±0.3	12.3±3.7	11.7±2	14.6±0.6
2015/10	K-fertilised	73.1±12.8	10.5±1.2	36.1±14.1	29.5±5.9	18.6±5.5
	Control	74.7± 8.6	10.9±0.5	41.2±13.8	32±9.1	18.1±3.9

Sample weights in August 2014 were obtained from pre-planted seedlings (n = 20), and the other weights were obtained from samples collected for radioactive analysis. Before weighting, all samples were dried at 75 °C for > 48 h. Each mean and SD was calculated from the mean of values at each plot (n = 4). There were no significant differences in heights, diameters and sample weights between the treatments (K fertilisation and control, $p > 0.05$, nested ANOVA).

Supplementary Table S2. Nutrient concentration (g kg^{-1}) in cypress needles sampled in October 2015.

Treatment	Na	Mg*	Al	P	K*	Ca*	Mn*	Fe	Zn
	[g kg^{-1}]								
K-fertilised	0.100±0.040	1.13±0.14	0.0618±0.0087	1.11±0.10	10.5±0.6	5.63±0.49	0.327±0.104	0.0718±0.0100	0.0262±0.0059
Control	0.120±0.041	1.45±0.07	0.0545±0.0135	1.14±0.14	8.75±0.66	6.67±0.46	0.434±0.076	0.0747±0.0151	0.0312±0.0029

The concentrations of elements in the needle samples were measured after wet digestion by HNO_3 and H_2O_2 (4:1) using ICP-MS (Agilent 7700x, Agilent Technologies). Each mean and SD was calculated from the mean of values at each plot (n = 4). Elements with asterisks (*) indicate that their concentrations were significantly different between the treatments ($p < 0.05$, nested ANOVA).

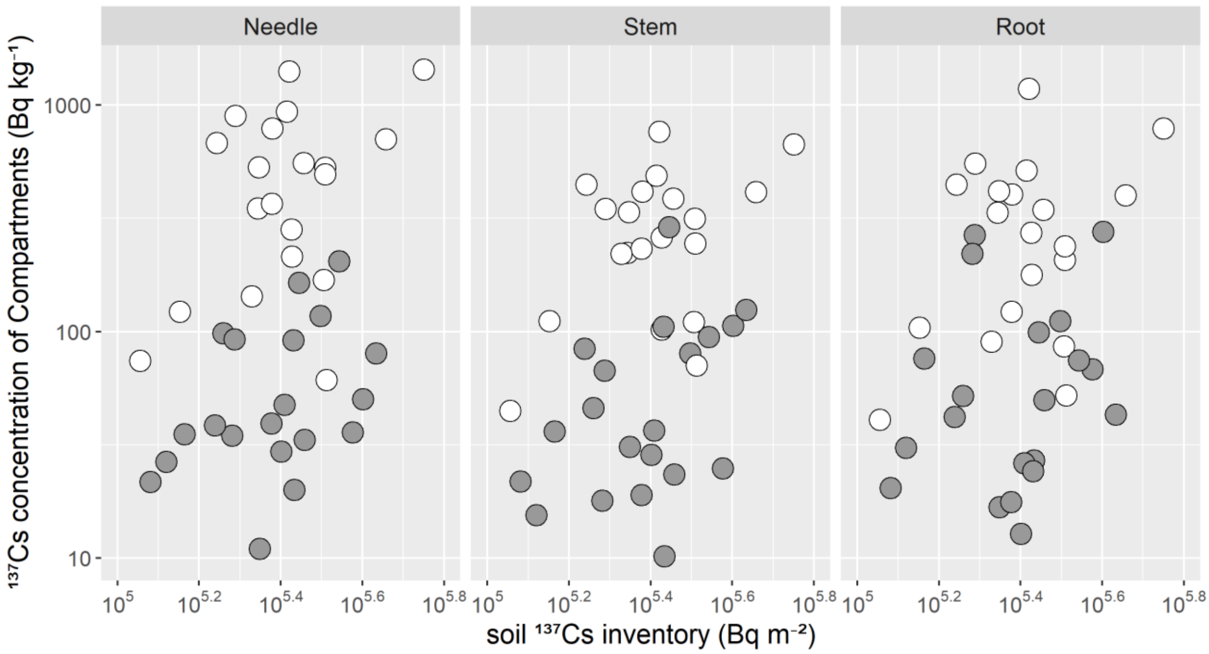

Supplementary Figure S1. ^{137}Cs concentrations in each compartment of seedlings against soil ^{137}Cs inventory sampled at control (open symbols) and K-fertilized (grey symbols) plots in October 2015 at the end of the second growing season. Logarithmic scales were used on the horizontal and vertical axes.
